# Effect of Load on Non‐Muscle Myosin 2 Paralog Filaments in a Biomimetic Contractile Actin Array

**DOI:** 10.1002/smll.202507772

**Published:** 2025-11-26

**Authors:** Philip Bleicher, David Han, Neil Billington, Ryan Hart, Christian A. Combs, Shureed Qazi, Indra Chandrasekar, Jay R. Knutson, James R. Sellers

**Affiliations:** ^1^ Laboratory of Molecular Physiology, National Heart, Lung and Blood Institute National Institutes of Health Bethesda MD 20814 USA; ^2^ Department of Biochemistry & Molecular Medicine, School of Medicine West Virginia University Morgantown West Virginia 26506 USA; ^3^ Enabling Technologies Group Sanford Research Sioux Falls SD 57104 USA; ^4^ NHLBI Light Microscopy Facility National Institutes of Health Bethesda MD 20814 USA; ^5^ Laboratory of Advanced Microscopy and Biophotonics, National Heart, Lung and Blood Institute National Institutes of Health Bethesda MD 20814 USA

**Keywords:** FRET, micropatterning, myosin, sarcomere, tension sensor

## Abstract

Cells express three non‐muscle myosin 2 (NM2) paralogs that form bipolar filaments of ≈30 motors each. Of these, NM2A and NM2B are best studied and show distinct enzymatic and mechanical properties. Although they can colocalize in cells, they also have unique localization patterns, suggesting functional differences. Most studies have examined these proteins interacting with actin under very low loads, but in cells they likely contribute to generating and maintaining cytoskeletal tension. Inspired by sarcomere‐like tension generation in non‐muscle cells, a minimal platform is reconstituted to study NM2 activity under load. Using micropatterned formin to align actin filaments in anti‐parallel bundles, phosphorylated NM2 filaments are introduced to observe their interactions via TIRF (Total Internal Reflection Fluorescence) microscopy. NM2B contracts and bundles the actin filaments to form a tensed system. The rate of myosin filament movement slows, and many filaments completely stall. In contrast, NM2A moved faster but disrupted bundles via severing and retraction. FRET‐based tension sensors revealed that both paralogs generate comparable tension despite these differing behaviors. This suggests that NM2A and NM2B play distinct mechanical roles in cells, with NM2B better suited for sustained tension and NM2A contributing to dynamic remodeling.

## Graphical abstract

The main paralogs of non‐muscle myosin 2 are studied on a micropatterned array of anti‐parallel actin filaments. This sarcomere‐like platform reveals myosin's load‐dependent behavior, and the resulting tension is quantified using embedded FRET sensors for both myosin and anchored actin. While the paralogs generate similar average tension, their distinct responses under load highlight differences in their mechanical roles.

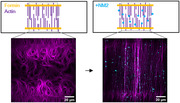

## 1 Introduction

Non‐muscle myosin 2 (NM2) paralogs, termed NM2A, NM2B and NM2C, are motor proteins and key mediators of actin cytoskeleton contractility.^[^ ^1^, ^2^ ^]^ They are essential for virtually all motility‐dependent cellular processes, such as migration, cytokinesis, or adhesion.^[^ ^3^, ^4^, ^5^ ^]^ Moreover, failures in NM2 paralog expression and regulation contribute to disorders like cancer, cardiovascular and neuronal diseases.^[^ ^6^ ^]^ Each NM2 molecule consists of two heavy chains which are unique to each paralog, two essential light chains (ELC) and two regulatory light chains (RLC), thus forming a hexamer. The tails form a parallel coiled‐coil dimer, creating myosin molecules that have two motor domains attached to a long tail. This hexameric structure is referred to as the myosin “monomer”.^[^ ^7^ ^]^ By anti‐parallel self‐association through their coiled‐coil tail domains, NM2 molecules form bipolar filaments^[^ ^8^ ^]^ with the two clusters of myosin motor domains separated by the anti‐parallel tail region. The filaments formed by NM2A or NM2B are each comprised of about 30 myosin monomers and are approximately 300 nm in length.^[^ ^9^ ^]^ In addition, the individual myosin filaments can self‐associate to form vertical “stacks” which sometimes further associate laterally to form elongated “daisy‐chain‐like” structures. The formation of stacks has been shown to be a dynamic process both in vitro and in vivo.^[^ ^10^, ^11^, ^12^, ^13^, ^14^ ^]^ Analysis of their kinetic and motile properties demonstrated that NM2A is about three times faster than is NM2B, but the latter has a larger duty ratio.^[^ ^10^ ^]^ The duty ratio is defined by the fraction of the kinetic cycle that the myosin spends in a state that is strongly bound to actin.

Given the polarity of the actin filament, a myosin filament can only move toward its barbed end.^[^ ^9^, ^10^ ^]^ This movement can be observed and quantified in an in vitro motility assay wherein fluorescently labeled myosin filaments can be seen to move along actin filaments bound to the surface.^[^ ^10^, ^15^ ^]^ This assay and most of the solution kinetic studies conducted with NM2 paralogs are conducted under conditions of zero or very low load. In cells, it is likely that these proteins are involved in mechanotransduction and generate and maintain force on actin filaments.^[^ ^16^ ^]^ We sought to develop a system where this mechanical behavior could be observed.

To this extent, we initially noted that NM2 localization in living cells was historically described as mostly isotropic.^[^ ^17^, ^18^ ^]^ However, this view has been challenged by the emergence of super‐resolution techniques in recent years. NM2 arrays have been found in vivo that show remarkable levels of organization such as along stress‐fibers, or just behind the leading edge of migrating cells.^[^ ^14^, ^19^, ^20^ ^]^ Another striking example is the near sarcomeric arrangement of NM2 at cell‐cell boundaries of hair cells, where NM2 and actin are localized in an alternating pattern along the network.^[^ ^14^, ^21^ ^]^ reminiscent of the fundamental tension‐generating sarcomeric structures found in muscle cells.^[^ ^22^, ^23^, ^24^ ^]^ Despite their relevance in cell motility and disease pathology,^[^ ^25^ ^]^ our understanding of highly organized, tension‐generating actomyosin structures in non‐muscle cells remains elusive. Consequently, the necessity to build experimental platforms that incorporate the force‐generation and mechanosensitivity^[^ ^26^ ^]^ of NM2 arises.

Here we address this issue by reconstructing tension generating structures of actin and NM2 filaments by using a multistep approach. Formin (FH1‐FH2 fragment of mDia1), a nucleator of actin filament assembly,^[^ ^27^ ^]^ is first bound to micropatterned, parallel lines on the coverslip. Upon addition of G‐actin and profilin, polarized actin filament elongation is initiated from each formin stripe resulting in elongated overlapping arrays of actin, anchored via their barbed‐ends. Regions between the stripes contain overlapping arrays of anti‐parallel actin filaments. These patterns mimic the inherently ordered actin structures found in non‐muscle cells. Following the establishment of this sarcomere‐like actin organization, bipolar NM2 filaments are added and their movement, along with that of the actin, is tracked using TIRF (Total Internal Reflection Fluorescence) microscopy. Our goal is not to study the function of sarcomere's per se, but rather to create a system wherein NM2 filaments are able to interact with ordered actin filament arrays of the opposite polarity. If the myosin slides these filaments toward the bare zone, then the filaments should straighten out and tense. Under these conditions, we can observe NM2 filaments operating under conditions of high internal load and observe their mechanical behaviors. In addition, we have used a FRET‐based tension sensor embedded into the myosin coiled‐coil structure to monitor the level of tension that the myosin is bearing via FLIM (Fluorescence Lifetime Imaging Microscopy). Similar FRET‐based probes have been calibrated previously and used to quantify tension with pN‐precision both in cells and in vitro.^[^ ^28^, ^29^, ^30^, ^31^, ^32^ ^]^ The same tension sensor embedded into the formin is used to examine the actin filaments’ tension as the myosin ensembles interact with them on the patterns. We conclude that the tension mediated by NM2 during network contraction is isotonic in nature, exerting a constant force averaging 4 pN/myosin with the majority of motor heads engaged simultaneously.

## 2 Results

**Figure 1:**
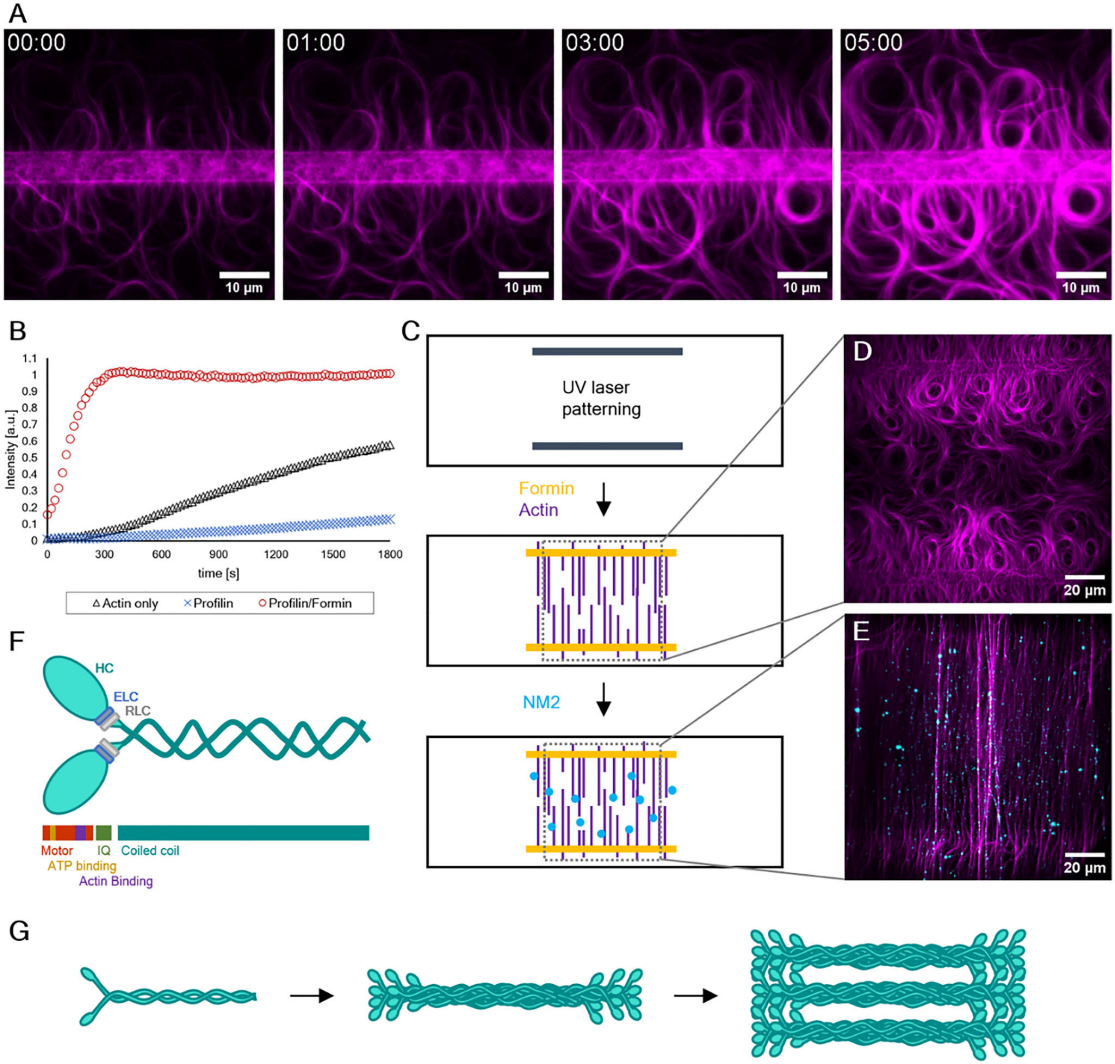
Reconstitution of a sarcomere‐like actomyosin assembly. A) Timeseries of TIRF images showing actin (magenta) filament polymerization perpendicularly away from micropatterned lines. The timestamps are depicted in minutes. B) Polymerization kinetics of actin monitored by pyrene fluorescence in the same buffer conditions (black curve). Profilin slows down spontaneous polymerization (blue curve). Formin catalyzed polymerization of actin (red curve) can be observed also in the presence of profilin. C) Micropatterning procedure. A UV laser is used to etch parallel lines into PEG‐silane passivated glass coverslips. Addition of formin allows its binding to the patterned regions. Excess formin is removed, then a polymerization mixture of profilin and actin is added. Addition of profilin minimizes spontaneous assembly of actin filaments, enabling efficient polymerization from patterned formin into an anti‐parallel actin array. In a final step, phosphorylated NM2 filaments are added to observe their motile effects on these structures. D) TIRF image showing actin after polymerization on patterned lines. E) Addition of NM2 (cyan) orients the actin filaments along the flow direction and leads to the formation of a tense, sarcomere‐like state. F) Cartoon representing the hexameric molecular structure of NM2, which is composed of two Heavy Chains (HC, turquoise), two essential light chains (ELC, blue), two regulatory light chains (RLC, grey). The heavy chains can be divided into a head and tail domain. The stick diagram shows NM2 heads contain the motor domain (red), ATP binding domain (yellow), Actin binding domain (magenta) and IQ domains which bind the LCs. The Tail domain contains the self‐interacting coiled‐coil. G) NM2 dynamically forms bipolar filaments through anti‐parallel self‐association via their tail domains. These filaments appear as punctae in TIRF microscopy. Filaments are further able to form multifilament stacks, appearing as brighter punctae of varying sizes.

To create highly ordered actin arrays, which mimic aspects of sarcomeric arrangement, parallel lines are micropatterned^[^ ^33^ ^]^ onto a passivated coverslip, then formin (using the truncated, constitutively active FH1 and FH2 domains of mDia1) is bound to these newly etched gaps in the passivation layer. Following the addition of G‐actin monomers and profilin, formin elongates actin filaments (purple) which extend out perpendicularly within five minutes (**Figure** **1**A). Formin‐independent background actin assemblies are suppressed by profilin (Figure 1B). Centered between the micropatterned lines, an anti‐parallel network of actin filaments forms, with the filaments’ pointed‐ends growing towards the center between the lines and the barbed‐ends bound to the immobilized formin (Figure 1C,D). Due to the presence of 0.6% methylcellulose, actin is crowded near the coverslip's surface and bundling can be observed (Movie S1, Supporting Information). These bundles move about as a result of thermal motion.

Upon addition of pre‐polymerized and phosphorylated NM2B (cyan) filaments to the assay, the myosin filaments can be observed binding to and moving along the actin arrays. The contractile activity of the myosins results in the shortening, linearization and further bundling of the actin filaments, leading to an array with a defined anti‐parallel orientation within 10 minutes after the addition of NM2B filaments (Figure 1C, E). In this assay the individual myosin filaments, which typically contain about 30 myosin monomers (Figure 1F) and are about 300 nm in length,^[^ ^9^ ^]^ appear as diffractionlimited puncta. When two or more myosin filaments get close, they coalesce into single spots which represent the stacks of filaments as has been shown previously^[^ ^10^ ^]^ (Figure 1G). This phenomenon has also been observed in live cell imaging experiments with labeled NM2 molecules.^[^ ^11^, ^13^, ^34^ ^]^ Thus, in our assay the myosin filament intensities vary accordingly to the number of myosin monomers in each filament and the number of filaments forming a stack (Figure 1E; Figure S1A–C, Supporting Information).

**Figure 2:**
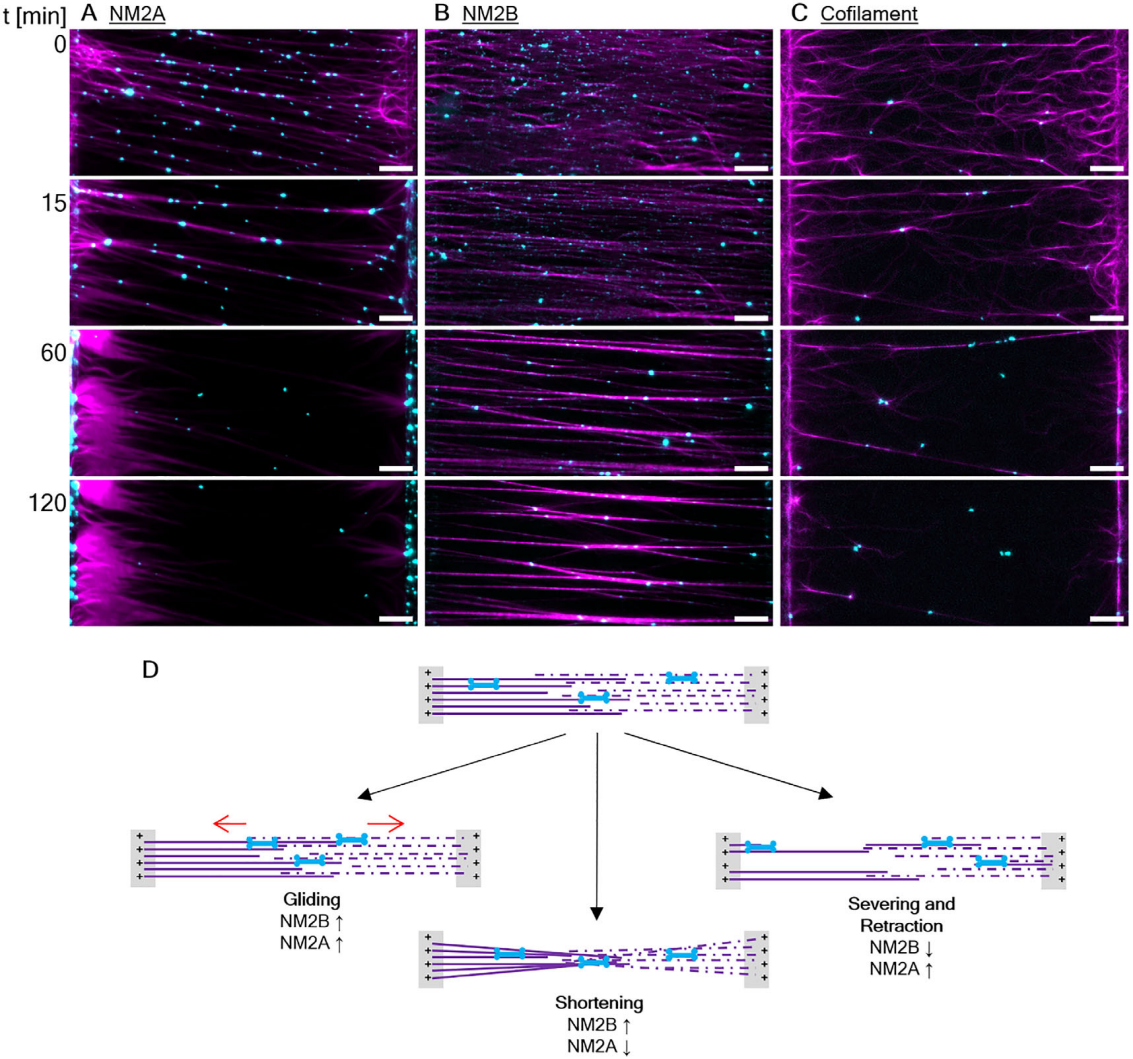
NM2 paralog dependent shortening in a sarcomere‐like actomyosin assembly. A) Time course of NM2A activity imaged by TIRF microscopy. Actin is shown in purple and NM2 is shown in cyan. The patterned lines are located along the left and right side of each frame. The distance between the patterned lines is 100 µm. The top images show the first imaged frames, 2 minutes after adding NM2 to the flow chambers. The time in minutes after the first image was taken is shown on the left. B) Time course of NM2B activity. C) Time course of equimolar cofilaments of NM2A and NM2B. Formation of tense bundles can be observed for both paralogs. In the case of NM2A, the actin bundles break and retract towards the barbed ends within 1 hour. The motility of NM2B filaments is generally slower and tense bundles remain for hours. Cofilaments show a tendency to disrupt the bundles similar to NM2A alone. Some bundles remain even after 1 hour, indicating that the ability to sever and retract is slowed down in cofilaments. D) Illustration summarizing the processes that can be observed during tension formation of the reconstructed sarcomere. The anti‐parallel orientation of the actin filaments (magenta) is indicated by solid (grown left to right) versus dotted lines (grown right to left), as well as by a “+”, as the barbed (+)‐ends are bound to the immobilized formin. Gliding of NM2 filaments is observed for both paralogs. Shortening and formation of tense bundles is the predominant process observed in the presence of NM2B, while NM2A favors rapid severing and retraction. All scale bars represent 10 µm.

A time course of the action of NM2B on the actin networks reveals that this is a slow process which is not unexpected given the slow actomyosin kinetics that has been described for NM2B^[^ ^35^ ^]^ (**Figure** **2**B). It takes more than 15 min for all the actin filaments to become bundled and tensed. After the initial contractile period the actin network remained largely stable for more than an hour, but there can be slow movement of the myosin filaments. Despite their initial processive motility, NM2B filaments stall as they are likely generating isometric tension on the straightened, anti‐parallel actin bundles. The myosin filaments display a tendency to accumulate in the center of the pattern (Figure S2, Supporting Information), where the overlap of the anti‐parallel actin networks is most pronounced (Movie S2, Supporting Information).

In contrast, NM2A filaments initially straighten the actin filaments, but also sever them. As the myosin filaments move towards the pattern line, they often pull the severed actin filaments with them, resulting in a depletion of actin filaments (and myosin) at the center of the pattern and accumulation of both NM2A and actin at the patterned line (Figure 2A). This all occurs in the first 20 min after myosin filament addition (Movie S3, Supporting Information).

Myosin 5a, a two headed motor capable of moving processively as a single molecule, moved along the actin filaments towards the patterned stripes where the actin barbed ends are located without contracting or shredding the actin filaments (Movie S4, Supporting Information). This indicates that a bipolar filament is required for sliding and tensioning, and in the case of NM2A, severing of the actin filaments.

**Figure 3:**
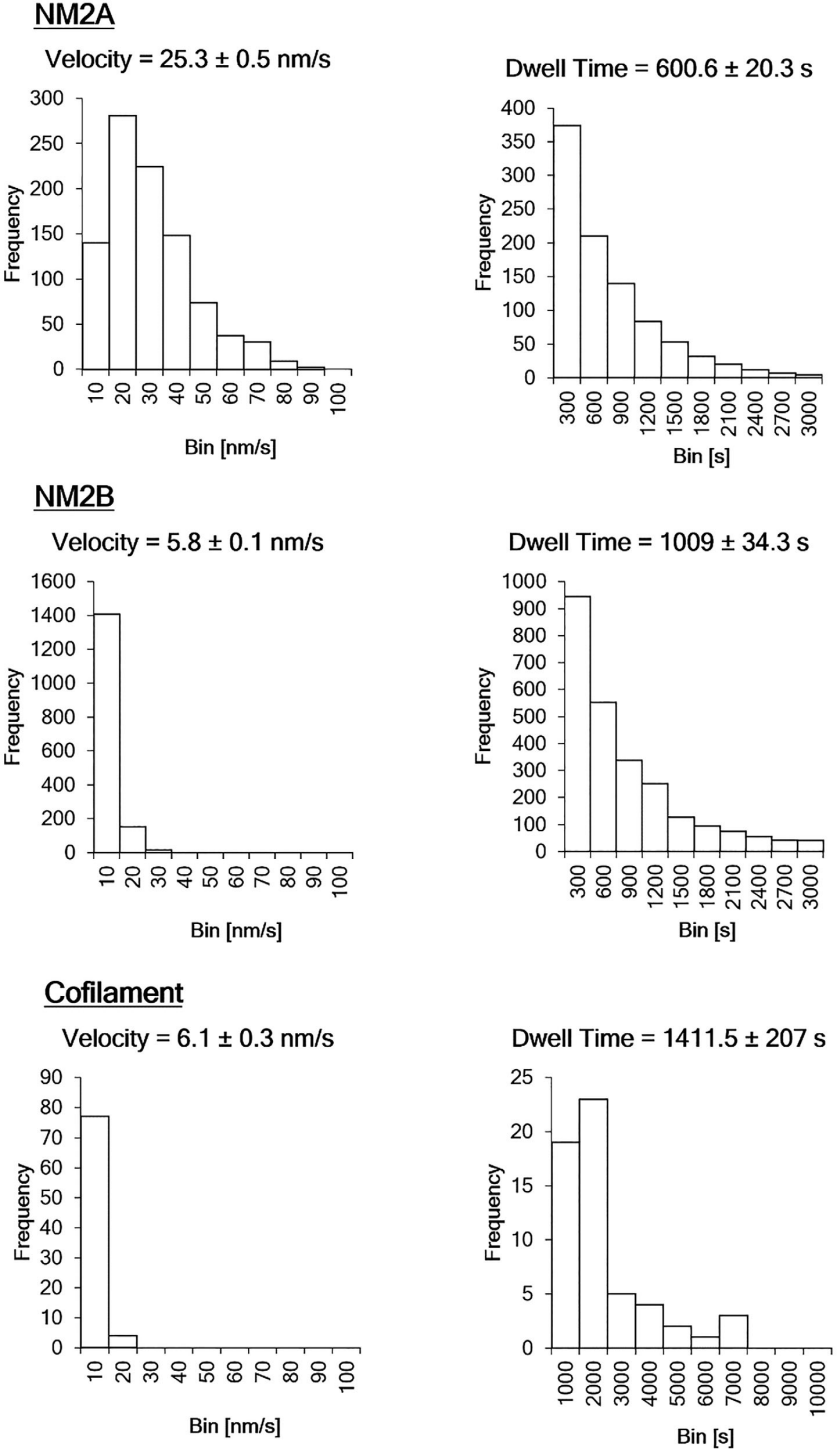
Motile properties of NM2 paralogs on sarcomeric actin patterns. A) Histograms of NM2A representing the NM2 filament's velocity (left) and dwell time (right). B) Respective histograms for NM2B. The average velocity of NM2A is 4 times higher. This difference is likely affected by their gliding activities, with NM2A's tendency to separate actin filaments. C) Respective histograms for equimolar cofilaments of NM2A and NM2B. All depicted errors are the distribution's standard errors.

Tracking and analysis of filament motility along the pattern reveals the motors’ average velocity until they stall, and the run eventually ends. The mean velocity of NM2A is more than 4 times greater than that of NM2B (**Figure** **3**A, B), consistent with the disparity found in measured velocities between the two paralogs on immobilized single actin filaments^[^ ^10^ ^]^ or in the gliding actin assay where individual filaments of actin are moved by myosin molecules bound to the coverslip surface.^[^ ^36^ ^]^ Although the difference between paralogs is retained, the magnitude of velocity of the filaments on the pattern is less than that observed with NM2 filaments moving on single actin filaments attached to the surface. In the latter case, the movement is likely to be largely unloaded and would be occurring at the maximal velocity for the particular paralog along unipolar actin filaments.^[^ ^10^ ^]^ The effect of load is also seen by the difference in dwell times of the myosin filaments on the patterns versus when they are moving on single actin filaments. The dwell time for NM2B filaments increases about 6‐fold on the patterns compared to when they are interacting with a single actin filament.^[^ ^10^ ^]^

It is known that NM2A and NM2B monomers can copolymerize both in vitro and in vivo^[^ ^2^, ^10^ ^]^ to form heterotypic filaments. Surprisingly, with cofilaments formed from an equimolar ratio of NM2A and NM2B, the shredding activity of NM2A on the pattern dominates, as most actin retracts and only a few bundles remain after 60 min (Figure 2C). In contrast, the motile kinetics of the cofilament are dominated by the slower paralog (Figure 3C) as was observed previously in motility assays with cofilaments moving on single actin filaments attached to a surface,^[^ ^10^ ^]^ resulting in a myosin ensemble that severs and retracts similarly to NM2A alone, but with the kinetics of the slower paralog.

In summary, NM2A rapidly shears actin filaments and moves processively toward the patterned line sometimes carrying severed actin filaments as cargo. In contrast NM2B addition to the patterns largely results in a stable pattern where myosin filament movement stalls due to simultaneous pulling on actin filaments of opposite polarity anchored to adjacent patterned lines. In this case the situation resembles a muscle sarcomere under isometric tension. This isometric tension becomes apparent when bundles are severed using laser ablation, which causes both severed ends to retract toward the patterned lines with a velocity of 14.5 nm s^−1^ for NM2A and 6.3 nm s^−1^ for NM2B (Figure S3, Supporting Information).

**Figure 4:**
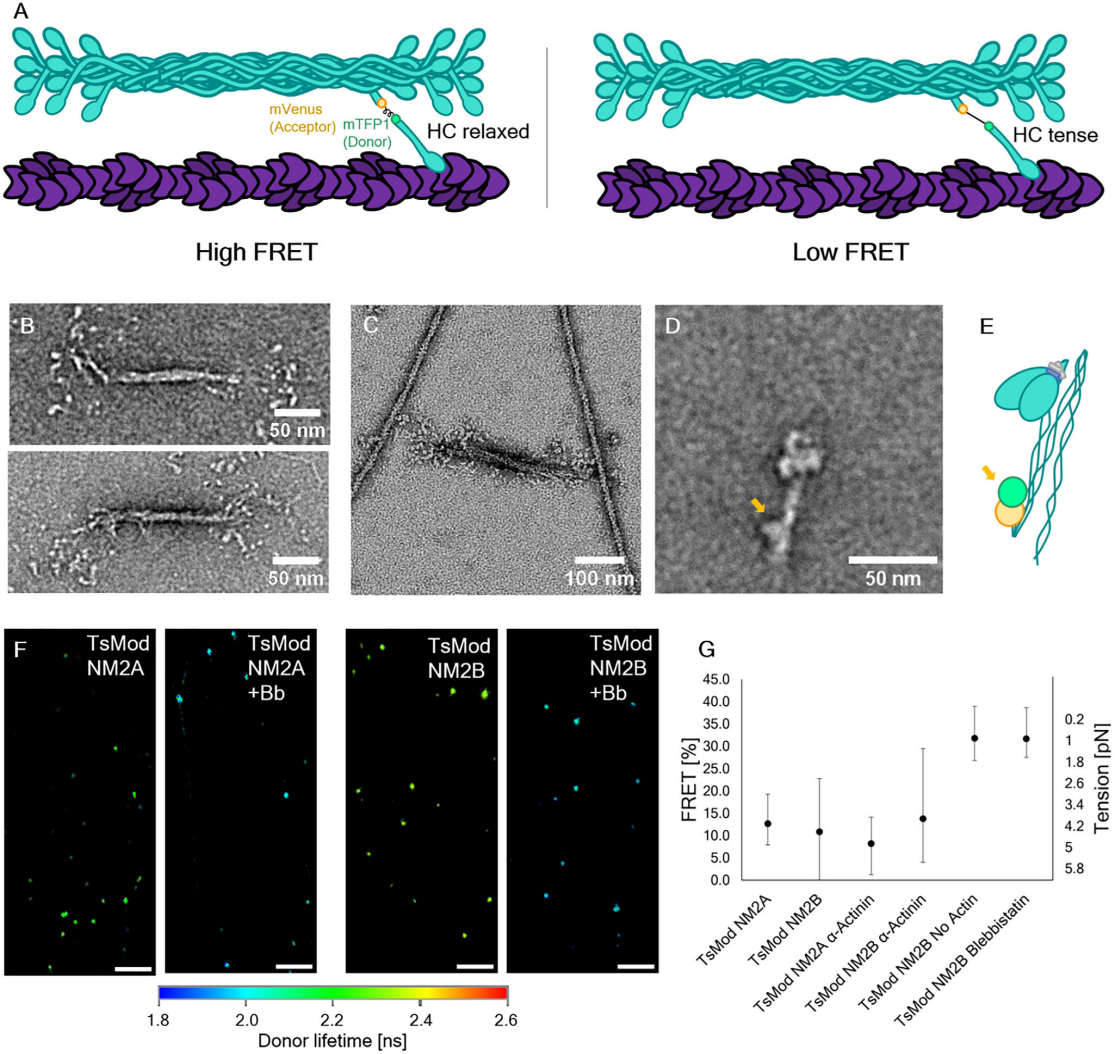
Force quantification using a FRET‐based NM2 tension sensor (TsMod NM2B). A) Illustration showing the construct design of the tension sensor. A FRET pair connected by a flexible linker is used to probe the tension of the heavy chain. In the relaxed state, the linker is shorter, and the FRET efficiency is higher. In the tense state, the linker gets stretched and the FRET efficiency is reduced. B) Electron microscopy demonstrates that the TsMod NM2B retains the ability to form bipolar filaments in the absence of ATP. C) Following phosphorylation of the RLC, TsMod NM2B myosin in the presence of ATP forms bipolar filaments which bind to actin filaments in the same manner as previously demonstrated for wild‐type NM2B.^[^ ^9^ ^]^ D) TsMod NM2B is able to form the 10S autoinhibited structure in the presence of ATP. The position of the FRET pair is visible in this state and marked with a yellow arrow. E) Illustration depicting the localization of the tension sensor in the 10S state (yellow arrow). Note that this cartoon only shows one of the FRET pairs, but in reality, the moiety is present in both heavy chains. F) FLIM image fits mapping the extracted long lifetimes to the donor wavelength's intensity. A region between two patterned lines with tense actin bundles was chosen. Both TsMod NM2A and TsMod NM2B show very similar lifetimes with no discernible tension gradient within one filament. Addition of para‐nitro‐blebbistatin (Bb) immediately shortens the Donor lifetime due to the relaxation of the linker and increased FRET. G) FRET efficiencies analyzed by global fitting of lifetimes with 90% confidence intervals. ROIs for fitting contained at least 10 individual myosin filaments and were confirmed in three independent experiments. The tension of NM2A and NM2B is indistinguishable. The addition of α‐actinin did not significantly affect the ability of NM2 to generate tension. NM2 filaments in the absence of actin or in the presence of para‐nitro‐blebbistatin report an increased FRET efficiency near 0 pN tension according to the sensor calibration.^[^ ^28^ ^]^ Note that due to the sensor's response ranging from 1‐6 pN, a FRET efficiency near 30% (or 1 pN) is probably the lowest possible tension that we can measure.

To probe the tension that an individual myosin is experiencing, we used a myosin with an embedded FRET‐based tension sensor module which was initially created for use in live cell imaging experiments.^[^ ^29^ ^]^ We will refer to this modified myosin as “TsMod NM2B”. The tension sensor consists of mTFP1 as a FRET donor and mVenus as acceptor, connected by a flexible linker. It was inserted at amino acid 1214 of NM2A's heavy chain, or amino acid 1224 of NM2B's heavy chain respectively, just past the S2 region and within the coiled coil. By stretching a flexible linker between the FRET pair, the FRET efficiency decreases, reporting tension as a function of FRET% (**Figure** **4**A). The questions arise as to whether this insert impairs myosin's enzymatic and mechanical properties, its ability to form bipolar filaments and whether it is still regulated by phosphorylation of the RLC.

To test whether the inserted module affects myosin structure or function we conducted several experiments. Negative staining electron microscopy demonstrates that unphosphorylated TsMod NM2B forms bipolar filaments that are indistinguishable from those of unmodified NM2B in the absence of ATP (Figure 4B). These filaments can bind to actin filaments after phosphorylation of the RLC and in the presence of ATP (Figure 4C). Unphosphorylated TsMod NM2B adopts the folded, monomeric, autoinhibited “off” state in the presence of ATP that was previously shown for unmodified myosin (Figure 4D). This implies that the phosphorylation‐dependent regulation is not impaired. The presence of the force sensor module can be seen as a bump near the point where the tail undergoes its back‐folding in averaged negatively stained images of TsMod NM2B in the presence of ATP (Figure 4D, E). Furthermore, the gliding motility of phosphorylated TsMod NM2 is within a factor of two compared to wildtype NM2 (Figure S4, Supporting Information).

Using this probe, the FRET efficiencies are determined using a Fluorescence Lifetime Imaging Microscope (FLIM). At least 10 NM2 punctae for both TsMod NM2A and TsMod NM2B were selected as ROI and combined with ROIs from at least three independent experiments per condition (Figure 4F). The intensity averaged lifetimes in each ROI are determined using a global fitting algorithm and the FRET% are calculated using the fluorescence lifetime of samples containing only donor fluorescence. Similar FRET efficiencies were found for both NM2A and NM2B, corresponding to a tension near 4 pN (Figure 4G). A FLIM image fit demonstrates the reduction of the donor lifetimes upon addition of 5 µM para‐nitro‐blebbistatin, a photostable inhibitor of NM2 which is functionally equivalent to blebbistatin (Figure 4G). Blebbistatin is known to inhibit the rate of phosphate release which leaves the myosin in a kinetic state that only weakly interacts with actin.^[^ ^37^ ^]^ The addition of α‐actinin, an actin binding protein which can cross‐link actin filaments of both parallel and anti‐parallel polarity, prevented network contraction (Movie S5, Supporting Information) and impaired myosin's motility (Figure S5, Supporting Information) but did not significantly affect the ability of individual myosin filaments to develop tension. As an additional control, samples without any actin were imaged and displayed a FRET efficiency near the lower detection limit of 1 pN.^[^ ^28^ ^]^ Additionally, a sensor with a dark acceptor was used as a zero‐FRET control, as well as a short‐linker version to force the fluorophores in a high‐FRET state was used to determine the lower and upper limit. This demonstrated that all lifetime measurements of contracted systems were within the sensor's range (Figure S6A,B, Supporting Information).

By using a global fitting algorithm, the amplitude of short and long lifetime components in each ROI could be determined. Assuming relaxed motors give rise to short lifetimes, while engaged motors contribute to the long lifetime components, these amplitudes revealed that a surprisingly high fraction of on average 80% of modules are tensed for both paralogs. This percentage slowly decreased during the experiments, which may be explained by the shortening of actin bundles and subsequent decrease of accessible actin filaments that NM2 can pull against (Figure S7, Supporting Information). The FRET module is located in the coiled‐coil region of each heavy chain, and it is likely that engagement of only one of the two heads of a given myosin would be sufficient to tense both of its FRET modules. Thus, the average of 80% tensed modules does not necessarily mean that 80% of the myosin heads in a filament are engaged with actin, but rather that at least 40% are. We also acquired FLIM fits of NM2 filaments gliding on single actin filaments, as opposed to patterned actin bundles, and determined that runs on isolated actin strands occur under virtually no load (Figure S8, Supporting Information).

**Figure 5:**
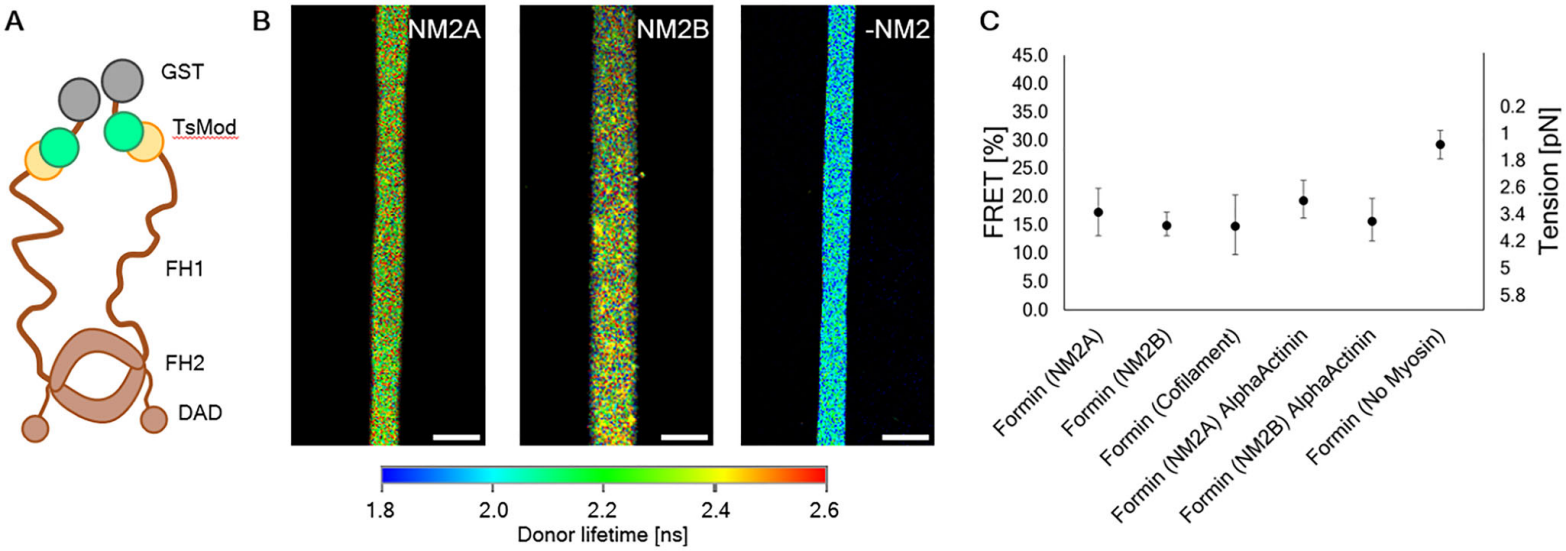
Quantification of the tension exerted towards the actin filaments by using a formin tension sensor. A) Illustration depicting the TsMod formin construct. Formin is N‐terminally truncated with an added GST‐tag followed by the TsMod, the G‐actin binding FH1 domain, F‐actin binding FH2 and regulatory DAD domain. B) FLIM image fits of the donor lifetime along patterned lines. Formin is indirectly immobilized on the patterned line by using a GST antibody. The same tension can be measured in the presence of either NM2 paralog. No tension is exerted in the absence of NM2, which is reflected by a shorter lifetime of the formin force sensor. C) FRET efficiencies analyzed by global fitting of lifetimes with 90% confidence intervals. ROIs for fitting contained at least 10 individual myosin filaments and were confirmed in three independent experiments. No significant difference can be observed for the FRET efficiencies of either paralog or cofilaments containing both. Adding α‐actinin to bundle actin filaments still leads to the same measured tension. In the absence of NM2 the FRET efficiency is increased to a tension closer to 0 pN according to the sensor's calibration. Note that due to the sensor's response ranging from 1‐6 pN, a FRET efficiency near 30% (or 1 pN) is probably the lowest possible tension that we can measure.

A TsMod formin was cloned to report the tension transmitted via individual actin filaments. A GST‐tag was added to the N‐terminal sequence of mDia1 to facilitate binding to the microfabricated stripes which were coated with an anti‐GST antibody (abcam, ab92) for these experiments. Next the TsMod force sensing motif was inserted before the start of the mDia1 FH1‐FH2 sequence. Following G‐actin addition, polar actin filament polymerization will ensue. The design of the construct ensures that the sensor gets stretched when tension is applied by myosin to the actin filaments tethered to the stripes by the anchored formin (**Figure** **5**A). Subsequently, the donor fluorescence can be observed in the patterned areas and the same tension near 4 pN can be found as reported for NM2, which could indicate that the force is directly transmitted from individual NM2 heads to actin filaments. However, it is possible that 4 pN is the maximal tension that is sensed by the module in this assay. Subsequently, at least 4 pN are transferred to actin filaments, while higher force averages cannot be detected. In the absence of NM2, the donor lifetime is reduced (Figure 5B). Fitting the data to determine the FRET efficiency shows that the tensions exerted by both paralogs and cofilaments of NM2A and NM2B appear to be the same, assuming the tension sensor is not maxed out. Additionally, α‐actinin was added to the networks as a crosslinker, to test a hypothesis that linking the actin filaments will lead to a cumulative effect regarding the mediated tension. However, no significant increase in tension was observed (Figure 5C).

Since the ability to build tension has been shown to be indistinguishable for both NM2 paralogs, other properties may be responsible for their respective behavior in sarcomeric actin arrays. For instance, a higher severing rate might explain NM2A's tendency to break and retract anti‐parallel bundles. We probe both NM2 paralogs' severing rates in this system by adding a 50 nM dose of covalently labeled rhodamine‐actin monomers to already polymerized patterns, in the presence or absence of NM2 filaments (Figure S9A,B, Supporting Information). The incorporation of the rhodamine‐monomers (red) as a fiducial marker into the previously polymerized network (green) should occur only at barbed ends, enabling the tracking of new ends that form via severing of actin filaments during the sarcomeric contraction. Using TIRF microscopy, we observe patches of rhodamine‐actin forming along the bundles and on retracted actin near the patterned lines. In the absence of NM2, there is no contraction of the lattice, and the incorporation of the secondary monomer (red‐actin) is drastically decreased (Figure S9C, Supporting Information). The intensity of rhodamine‐monomers plotted against time shows the total incorporation for both paralogs and in the absence of NM2 (Figure S9D, Supporting Information). To quantify severing rates, the incorporation of red actin in the absence of NM2 is subtracted, leaving only the contribution of NM2‐mediated actin incorporation. This revealed a rate of 0.071 min^−1^ for NM2A and 0.036 min^−1^ for NM2B via single exponential fitting. The higher rate for NM2A is consistent with its faster kinetics compared to NM2B.^[^ ^38^ ^]^ However, both paralogs can induce nicks within the actin bundle, supporting the idea of NM2's role as an enhancer of actin filament dynamics via barbed end formation.^[^ ^12^, ^39^, ^40^ ^]^

## 3 Discussion

The movement of NM2 filaments along single unipolar actin filaments bound to a surface has been studied previously. These studies showed that filaments of NM2B move more processively but three times more slowly than do filaments of NM2A.^[^ ^10^ ^]^ In these studies, the filaments should be moving under conditions of very low load. In order to examine the movement of NM2 filaments under conditions where they are experiencing loads and generating internal forces, we used micropatterning to create arrays of actin filaments of opposite polarity where each actin filament was bound via its barbed end to the surface via the formin that initiated its polymerization. Addition of preformed phosphorylated NM2 filaments to these patterns revealed that they initially bind randomly. In homology with the longer bipolar myosin filaments of striated muscle and in agreement with electron microscopic images of NM2 filaments in vitro,^[^ ^9^ ^]^ it would be expected that a given bipolar NM2 filament would be capable of simultaneously interacting with oppositely polarized actin filaments and sliding both of them toward the center of the myosin filament. While this array bears some resemblance to a muscle sarcomere, it was not our intention to use this system to investigate the function of the less ordered and more closely spaced sarcomeres that exist in muscle cells. In particular, it should be noted that unlike muscle sarcomeres, the spacing between the actin binding sites is fixed and does not contract. The spacing between the actin‐attachment points is larger than that found in vertebrate muscle sarcomeres and in some non‐muscle cells. In addition, muscle sarcomeres contain an abundance of structural and regulatory proteins such as titin, myosin binding protein‐C, nebulin, troponin and tropomyosin, which we have not attempted to replicate.

In our reconstructed system, NM2B filaments move along the actin filaments which are anchored to one or the other parallel strip while sliding the actin filaments of opposite polarity resulting in a shortening and drawing them into tight, linear bundles. This process is slow, reflecting the slow actomyosin kinetics of this motor, but the tensed actin filaments remain stable for several hours. The motile properties of NM2B filaments on these bipolar actin arrays differ from those observed when moving on a single unipolar actin filament. The velocity is reduced, and the lifetime of attachment increases dramatically. The average dwell time is more than fifteen minutes. Measurements of run length are not informative, since many of the myosin filaments stall during this period, likely as a result of the balanced forces being generated by motors interacting with anchored, oppositely polarized actin filaments; the attachment time is a better parameter to describe the kinetics. These behaviors would be expected if the release of ADP from the actomyosin cross bridge is force sensitive. Such force sensitivity of the attachment lifetime has been shown for single smooth muscle myosin molecules using optical trapping.^[^ ^41^, ^42^ ^]^ ADP release from the AM.ADP state is the kinetic step that limits the attachment lifetime. Kovács et al.^[^ ^26^ ^]^ showed that both NM2A and NM2B paralogs released ADP much more slowly when the cross‐bridge was under strain which would resist the ability of the cross‐bridge to complete its powerstroke. Of the two paralogs NM2B was the most force‐sensitive. Since the myosin. ADP kinetic intermediate binds strongly to actin, this would tend to slow the rate of cross‐bridge cycling and increase the number of myosin motors attached to the filaments at any instance. At the extreme of this behavior, the myosin. ADP cross‐bridge would form a catch‐bond with actin, meaning that the actomyosin interaction is stabilized by the exerted tension. Within a cellular sarcomere‐like organization, this would allow for the maintenance of tension with very little expenditure of energy.

The behavior of NM2A filaments on these patterns is strikingly different. Upon addition of NM2A filaments to the preformed patterns the actin filaments are broken and are collapsed back toward the stripes. NM2A filaments can be seen translocating towards the stripes and in some cases are carrying multiple short, shredded filaments with them. As expected from its more rapid actomyosin kinetics, NM2A filaments moved about four times faster than those of NM2B in these assays.

Note that these two extreme behaviors exhibited by the two NM2 paralogs may reflect their intracellular functions. NM2B exists primarily in stress fibers where it likely functions to maintain steady tension on oppositely oriented actin filaments.^[^ ^43^ ^]^ NM2B remains strongly attached to actin for about 3 seconds during a cross‐bridge cycle under conditions of no or very low load as determined by transient kinetic studies as well as optical trapping.^[^ ^15^ ^]^ The force dependence of its kinetics observed here and in the aforementioned transient kinetic^[^ ^38^ ^]^ study suggest that under these loaded conditions the ATPase activity would be markedly suppressed and that a myosin would be able to remain in a force bearing, actin‐attached state longer than a minute for each ATP that was hydrolyzed resulting in a remarkable conservation of energy.

The lamellipodia of many cell types contain a branched mesh work of actin filaments formed by the Arp2/3 actin nucleator. Just behind this region is an actin bundle that lies parallel to the leading cell edge. NM2A is often localized very prominently just behind the Arp2/3 network and in the actin bundle.^[^ ^40^ ^]^ We showed that both paralogs of NM2 are very effective bundlers of actin filaments. In addition, it has been proposed that a major function of NM2A in this region might be to shred actin filaments to create more ends in order to accelerate the overall depolymerization rate of the population to create more G‐actin monomers to feed the polymerization machinery that drives the propulsion of the leading edge of the cell.^[^ ^40^ ^]^ Our studies demonstrate that NM2A is very efficient at doing this. Why NM2A is more effective at severing actin filaments is not known. There are no high resolution cryoEM studies of NM2A and NM2B bound to actin in the myosin.ADP or apo states and so comparisons of their binding sites cannot be made at this time. It is possible that the powerstroke of NM2A imparts a torque onto the actin filament that would not occur with NM2B.

Aside from kinetic information, another parameter accessible in this system is the tension borne by NM2 due to the formation of prolonged contractile structures. Note that when using the myosin tension sensor, the average lifetimes we measure result from signals of single motors in ensembles. Similarly, in the assay using the formin tension sensor, we are only measuring the tension generated on a single actin filament and not the sum total of the tension generated on the pattern. However, even when actin filaments were bundled by α‐actinin, no increase in the measured tension was observed. One possible explanation is that NM2 motors are unable to produce tension on an actin filament higher than 4 pN. However, a possible limitation of the FRET sensor is that it may be unable to report higher tension in our ensemble assays, meaning that 4 pN may be the detection limit instead. Although we cannot exclude this possibility, our controls for the minimum and maximum FRET range made in the same conditions (Figure S6A,B, Supporting Information) suggest that all measured donor lifetimes are well within the sensor's range.

Surprisingly, the measured tension with the myosin sensor was constant while contracting and as such, NM2 tension is isotonic in nature, but not truly isometric as sliding of actin filaments and shortening of bundles is still possible, unless prevented via additional crosslinkers. A likely explanation for this ability is the dual nature of NM2 filaments to act as crosslinker and processive motor, enabling it to build tension via its multiple heads without eventually collapsing the tension of the associated actin network due to stress‐induced nicking. Thus, it fulfills the opposing demands of tension mediator and actin dynamics enhancer through barbed end formation, albeit to a different extent depending on the paralog. While NM2A is four times faster and has a twice as high severing rate, NM2B can form persistent bundles that are held together by its own crosslinking, effectively acting as a clamp. Both paralogs may as well act as a catch‐bond as described previously, which could explain their tendency to hold on to the actin bundles after contraction.

Optical trapping has demonstrated a similar catch‐bond mechanism for skeletal muscle myosin.^[^ ^44^ ^]^ The study demonstrated that the lifetimes of actomyosin interactions are highly load‐dependent for forces up to 6 pN, which is very close to the maximal force produced by a single skeletal muscle myosin 2 molecule. Given its adaptation for rapid contractions embedded in sarcomeres, we speculate that NM2, on the other hand, may operate at lower loads, suitable for the dynamic mechanical requirements found in non‐muscle cells.^[^ ^45^, ^46^ ^]^ Future single molecule studies could confirm whether NM2's load‐dependence and maximal force are indeed around 4 pN, which is a limitation that cannot be addressed by the ensemble measurements using the FRET sensor.

Another noteworthy difference of the paralogs is a surprisingly high variance of TsMod NM2B's tension, compared to the tension formed by TsMod NM2A. A possible explanation for this disparity is the inherent dynamics, as FLIM ensemble measurements are averaged over several minutes and ROIs combining the data from several filaments per ROI. This variance likely results from fluctuating tension due to the dynamic cycling of NM2 heads. In contrast, the TsMod formin displayed a much smaller variance in the presence of either paralog. It is surprising that formin's average tension never exceeded 4 pN, considering that for skeletal muscle myosin 2, ensembles of motors have been shown to generate force cooperatively and exceeding the force of single motors.^[^ ^47^, ^48^, ^49^ ^]^ Notably, formin's polymerization rate are directly dependent on the applied load and already much lower forces of 0.4 pN produce a dramatic increase.^[^ ^50^ ^]^ This suggests that formin‐polymerized actin filaments that are under NM2‐mediated tension are at least 10 times above the threshold for optimal growth in cells.

Taken together, this reveals NM2 as an isotonic tension generator, with distinct paralog specific properties. Since the magnitude of tension generated by each myosin paralog is very similar and both can break actin filaments to form barbed‐ends via stress‐induced actin nicking, their striking differences is likely a kinetic phenomenon. Notably, the cofilament which kinetically coincides with the slower paralog NM2B was unable to form tense bundles to the same extent as NM2B and displayed NM2A's tendency to retract actin without stalling. On the other hand, the paralog's distinct behavior may be explained by load‐dependent bond types, as motors under tension may have an increased tendency to slip or bind respectively, depending on the paralog's adaptation. This hypothesis could be confirmed via optical trapping or in silico in further studies.

## 4 Experimental Section

### Vector Construction

TsMod NM2A and NM2B, as well as the 5AA, DA and DD controls were kindly provided by Dr. Indra Chandrasekhar. TsMod formin, 5AA, DA and DD were cloned via overlap extension PCR using the primers TsMod_f_BamHI (5′‐ GATCGGATCCATGGTGAGCAAGGGCGAGGAGACCACAATGGGCGTAAT‐3′) and TsMod_r_Linker (5′‐ATCACTCTCGGCATGGACGAGCTGTACAAGCTGGTGCCGCGCGGCAGC‐3′) for the tension sensor module fragment, and mdia1_f_Linker (5′‐CTGGTGCCGCGCGGCAGCATGGCTTCTCTCTCTGCTGTGGTTGTTGCA‐3′) and mdia1_r_NotI (GTGCAAGCGGGCATCATCATCATCATCATCATCATTAAGCGGCCGCGTAC) for the formin fragment, then subsequently cloned into pGEX‐6P‐1.

### Protein Purification

Rabbit skeletal actin was purified as described previously^[^ ^51^ ^]^ and G‐actin monomers were lyophilized for storage at ‐20 °C. Fresh aliquots of monomeric actin were dialyzed against G‐Buffer (2 mM Tris, 0.2 mM ATP, 0.2 mM CaCl_2_, 0.2 mM DTT and 0.005% NaN_3_, pH 8.0) and used for up to one week. Formin mDia1 and TsMod mDia1 were purified as described previously.^[^ ^52^ ^]^ Halo‐tagged NM2s and TsMod NM2s were purified, phosphorylated and polymerized according to a published protocol.^[^ ^53^ ^]^ The GST antibody was purchased from Abcam, all other proteins were purchased from Cytoskeleton Inc.

Micropatterning and sample preparation. Sample chambers were prepared using 1.5H glass coverslips (25 mm x 75 x mm) purchased from ibidi. Coverslips were first washed rigorously with water and ethanol, then dried in air flow, before treatment in a Zepto plasma cleaner (Diener electronic, Germany) using argon plasma for 5 min. Coverslips were coated with 2 mg mL^−1^ mPEG (MW 2000), then dried and stored at ‐80 °C for further use. The sample chambers were then assembled using a stored PEG coverslip and a sticky‐Slide VI 0.4 (ibidi).

Micropatterning of parallel lines with 100 µm distance was performed using a PRIMO (alveole) and PLPP classic solution (alveole) with a laser dose of 1700 mJ mm^−2^. Channels used for patterning were then washed 3 times with PBS and either stored for up to one day or used directly. Actin patterns were prepared by adding 2 µM mDia1 in PBS directly to the flow channels, or using a GST antibody (abcam, ab92) to bind mDia1 via its N‐terminal GST‐tag. After allowing the proteins to bind for 5 minutes, the channels were washed 3 times with PBS, then 3 times with KMEI (50 mM KCl, 1 mM MgCl_2_, 1 mM EGTA, 10 mM Imidazole HCl, pH 7.0).

Perpendicular elongation of actin filaments was achieved by flowing in an actin polymerization mix containing 2 µM G‐actin, 10 µM profilin, 1 mM ATP, 1 mM DTT, 1 µM rhodamine‐Phalloidin (Thermo Fisher) and 0.6% Methylcellulose in KMEI. Additionally, a glucose oxidase/catalase scavenger (2.5 mg mL^−1^ glucose, 100 µg mL^−1^ glucose oxidase, 40 µg mL^−1^ catalase) was implemented to monitor polymerization via TIRF microscopy. The polymerization was incubated for 30 min at room temperature to allow completion. Afterwards, the channels were washed 3 times in KMEI, then 3 times in MB150 (150 mM KCl, 5 mM MgCl_2_, 0.1 mM EGTA, 20 mM MOPS, pH 7.4).

Phosphorylated filaments of NM2 were diluted to 50 nM in a motility solution containing final concentrations of 1 mM ATP, 1 mM DTT, optional 50 nM Alexa Fluor 488 halo ligand (Promega) for halo‐tagged NM2, 0.6% Methylcellulose in MB150 with a glucose oxidase/catalase scavenger. The chambers were then immediately used for imaging. Para‐nitro‐blebbistatin was purchased from Cayman Chemical (Item No. 24 171).

### Imaging and Data Acquisition

Electron microscopy of NM2 filaments was performed at the NHLBI's Electron Microscopy Core as described previously using a published method.^[^ ^9^ ^]^

TIRF microscopy was performed on an inverted Nikon Eclipse Ti‐E microscope with an H‐TIRF module, a CFI60 Apochromat TIRF 100X Oil Immersion Objective Lens (N.A. 1.49, W.D. 0.12 mm, F.O.V 22 mm) and an EMMCD camera (Andor iXon Ultra 888 EMCCD, 1024 × 1024 array, 13 µm pixel). The sample temperature was kept at 30 °C using a stage top incubator (TOKAI HIT, Japan). A 488 nm laser was used to image halo‐NM2 labeled with an Alexa Fluor 488 Halo ligand (Promega) or TsMod NM2, or actin stabilized with Alexa Fluor 488 Phalloidin (Thermo Fisher), and a 561 nm laser was used for actin stabilized with Rhodamine Phalloidin (Thermo Fisher).

Fluorescent lifetime imaging (FLIM) was performed using a Leica Falcon SP8 microscope, a pulsed white‐light laser tuned to 470 nm, a Leica HyD SMD hybrid photomultiplier tube observing quenched donor emission between 480 and 520 nm, and a Leica HC PL APO CS2 63x, 1.40 NA oil immersion objective (Leica Microsystems, Inc., Wetzlar, Germany). All images were taken with a scanning speed of 400 Hz, a pixel format of 512x512, and a pinhole size of 1.0 AU. Prior to imaging, the laser power was adjusted to obtain fewer than 0.1 photons/laser pulse (approximately 5 µW at the back aperture). To ensure adequate photons were collected per pixel, 100 frames were obtained per image.

### Data Analysis

Regions of interest were selected in the FLIM images according to their likely ability to provide varying amounts of the constituent lifetimes, i.e., expected mixture of high versus low tension. Photon counts versus time for pixels in these ROIs were aggregated to produce ROI‐specific decay curves, which were subjected to temporal reconvolution and global analysis to recover the consensus lifetimes (donor lifetimes which, in turn, map to differing extensions and hence tensions). Global nonlinear least‐squares analysis of this type was designed to accurately recover consensus parameters like lifetime by both reducing the number of parameters sought from data and by restricting parameter covariance while fitting.^[^ ^54^ ^]^ The amplitudes associated with each of these global lifetimes represent molecular populations, hence the average lifetimes gleaned from these fits represent the concentration‐weighted mixture of tension levels. The rigorous 67% and 90% confidence limits on both the lifetimes and those population levels were further derived using “support plane” analysis and the F‐test.^[^ ^55^, ^56^ ^]^ In nonlinear least squares, error bars derived this way were not necessarily symmetric (+/−).

## Conflict of Interest

The authors declare no conflict of interest.

## Author Contributions

P.B. and J.R.S. designed the research. P.B., D.H., N.B., and C.A.C. performed the experiments. R.H. and I.C. designed the original myosin tension sensor. P.B., S.Q., and J.R.K. analyzed the data. P.B. and J.R.S. wrote the main manuscript text. All authors discussed the results and revised the manuscript.

## Supporting information



Supporting Information

Supplementary Movie S1

Supplementary Movie S2

Supplementary Movie S3

Supplementary Movie S4

Supplementary Movie S5

## Data Availability

The data that support the findings of this study are available from the corresponding author upon reasonable request.
