# Supplementary material for: Effect of Load on Non‐Muscle Myosin 2 Paralog Filaments in a Biomimetic Contractile Actin Array
Source: Small. 2025 Nov 26;22(3):e07772. doi: 10.1002/smll.202507772 (PMC12802541; doi:10.1002/smll.202507772)
Supplement: Supplementary file 1 — Supporting Information [file SMLL-22-e07772-s005.pdf]

NANO · MICRO
small

Supporting Information

for *Small*, DOI 10.1002/smll.202507772

Effect of Load on Non-Muscle Myosin 2 Paralog Filaments in a Biomimetic Contractile Actin Array

Philip Bleicher, David Han, Neil Billington, Ryan Hart, Christian A. Combs, Shureed Qazi, Indra Chandrasekar, Jay R. Knutson and James R. Sellers**

Supplementary Information

Effect of Load on Non-Muscle Myosin 2 Paralog Filaments in a Biomimetic Contractile Actin Array

Philip Bleicher[1,*], David Han[1], Neil Billington[1,2], Ryan Hart[3], Christian A. Combs[4], Shureed Qazi[5], Indra Chandrasekar[3], Jay R. Knutson[5], James R. Sellers[1,*]

[1]Laboratory of Molecular Physiology, National Heart, Lung and Blood Institute, National Institutes of Health, Bethesda, MD, USA

[2]Department of Biochemistry & Molecular Medicine, School of Medicine, West Virginia University, Morgantown, West Virginia, USA

[3]Enabling Technologies Group, Sanford Research, Sioux Falls, SD, USA

[4]NHLBI Light Microscopy Facility, National Institutes of Health, Bethesda, MD, USA

[5]Laboratory of Advanced Microscopy and Biophotonics, National Heart, Lung and Blood Institute, National Institutes of Health, Bethesda, MD, USA

*Corresponding Author

Supplementary Movie S1. Polymerization of actin bundles from parallel, patterned lines. Formin is covalently bound to the patterned lines and a polymerization solution containing monomeric actin, profilin and rhodamine-phalloidin (purple) is added to initiate the actin elongation. As actin's barbed ends are attached to the patterned formin, the pointed ends extend towards the center area, mimicking the orientation of actin found in sarcomeres. Due to the overcrowding, actin bundles start forming loops and the presence of 0.6% methylcellulose causes bundling.

Supplementary Movie S2. NM2B (cyan) contracts sarcomeric actin (purple), forming long-lasting, tense bundles. Addition of the motility solution straightens the actin filaments along the flow direction and allows myosin filaments to attach to the actin. Gliding towards the patterned lines can be observed, which is where actin's barbed ends are located, as well as tension formation as bundles are stable for the course of 2 h in the presence of NM2B.

Supplementary Movie S3. NM2A (cyan) contracts sarcomeric actin (purple) by dragging actin to the patterned lines where the barbed ends are located. Unlike NM2B, bundles are much less stable in the presence of NM2A due to severing and retraction.

Supplementary Movie S4. Myosin 5a (cyan) moves glides on sarcomeric actin (purple) processively without altering the actin structure. Due to its two-headed structure, gliding towards the barbed ends located at the patterned lines only leads to actin fluctuation, while no contraction or retraction of actin can be observed..

Supplementary Movie S5. NM2A (left, cyan) and NM2B (right, cyan) on sarcomeric actin patterns (purple) crosslinked by α-actinin. On crosslinked actin, NM2's motility is reduced and contraction and severing of the bundles is prevented.

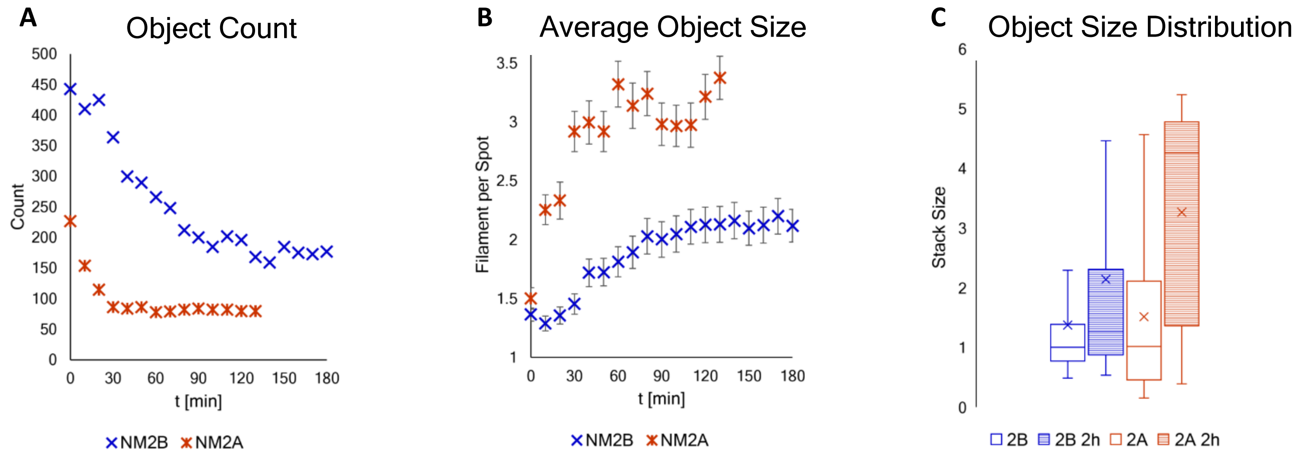

Supplementary Fig. 1: Stack formation of NM2 paralogs in sarcomeric arrays.

A By counting the number of myosin punctae along the pattern ("object count"), the coalescence of stacks can be quantified, as spots merge into each other. A representative pattern was analyzed for both paralogs and only spots between the patterned lines were counted. NM2A stacks are fully coalesced after 30 minutes, while NM2B's stack count only plateaus after 90 min. As virtually no detachment of NM2 filaments and stacks from patterns can be observed, the decline in spot counts is likely due to filament coalescence only. **B** By dividing the spots total intensity by the median intensity of single myosin filaments, their stack sized can be determined. On average, NM2A's reaches stacks of 3 filaments, while NM2B averages on 2 filaments per stack. **C** The distribution of this stack size is shown with a box plot, showing that in their contracted state after 2 h, NM2A shows a much wider distribution, with stacks containing up to 5 filaments.

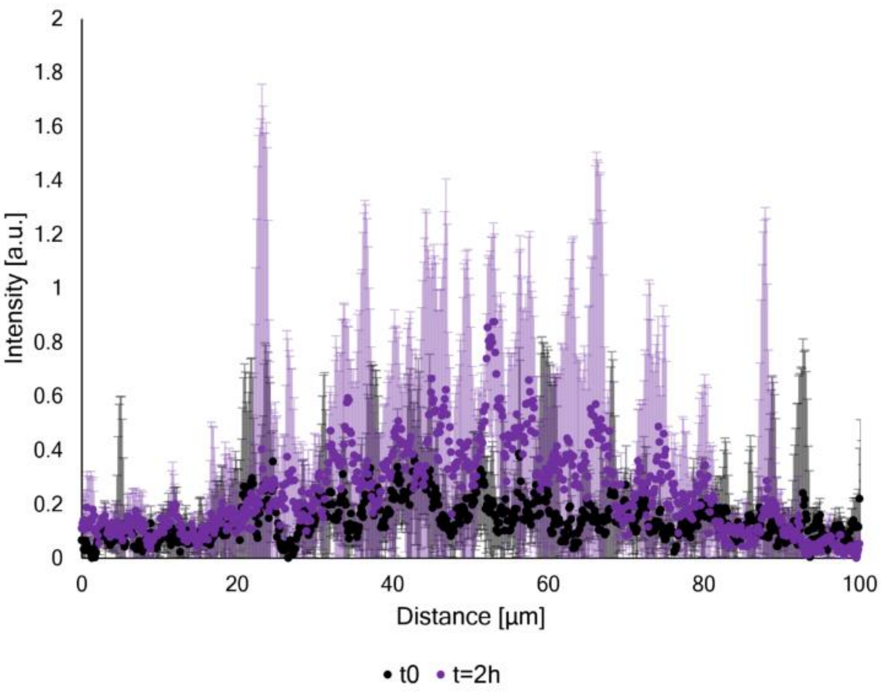

Supplementary Fig. 2: Myosin intensity distribution along sarcomeric actin bundles.

The intensity of NM2 filament fluorescence is measured in ROIs between two patterned lines and the x-axis represents the 100 µm distance between both lines. The intensity values are averaged and collected from three individual patterns, the error bars are their standard deviation. The intensity values from right after adding NM2 filaments to the pattern (t0) is shown in black, and the state after 2 h of contraction is shown in purple. NM2B displays a tendency to accumulate at the center region between the lines. This is likely due to actin's architecture, as the overlap between the long actin filaments is most anti-parallel in the middle of the pattern.

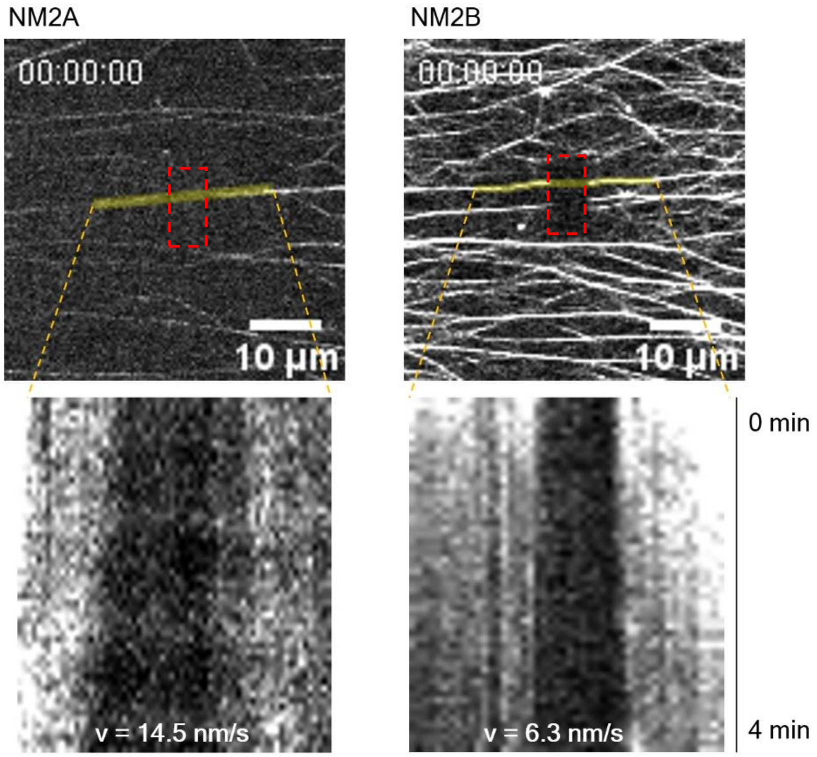

Supplementary Fig. 3: Laser ablation of tensed sarcomeric actin and subsequent retraction of severed bundles.

Using the Leica LAS X FRAP software, a rectangular ROI (red dashed lines) is selected, then ten iterations of a 1 mW UV laser dose is applied in the bleaching step. This was enough to visibly sever a tense actin bundle of a sarcomeric pattern, leading to retraction of the two severed ends. The retraction speed is faster for NM2A at around 14.5 nm/s, and slower for NM2B at 6.3 nm/s. Severed bundles get retracted towards the patterned lines, which is shown via a kymograph for the actin bundle. The kymograph is tracking the yellow highlighted part of the severed bundle.

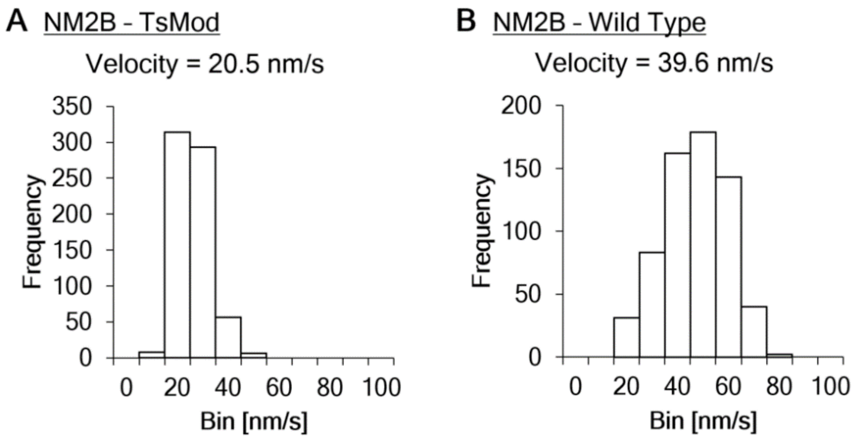

Supplementary Fig. 4: Motile properties of TsMod NM2B compared to wild type NM2B using the gliding actin motility assay.

By immobilizing single NM2B molecules on a glass surface, their ability to move labeled actin are studied in an actin gliding assay. **A** NM2B TsMod slides actin at an average velocity of 20.5 nm/s **B** while the wild type displays a typical velocity of 39.6 nm/s.

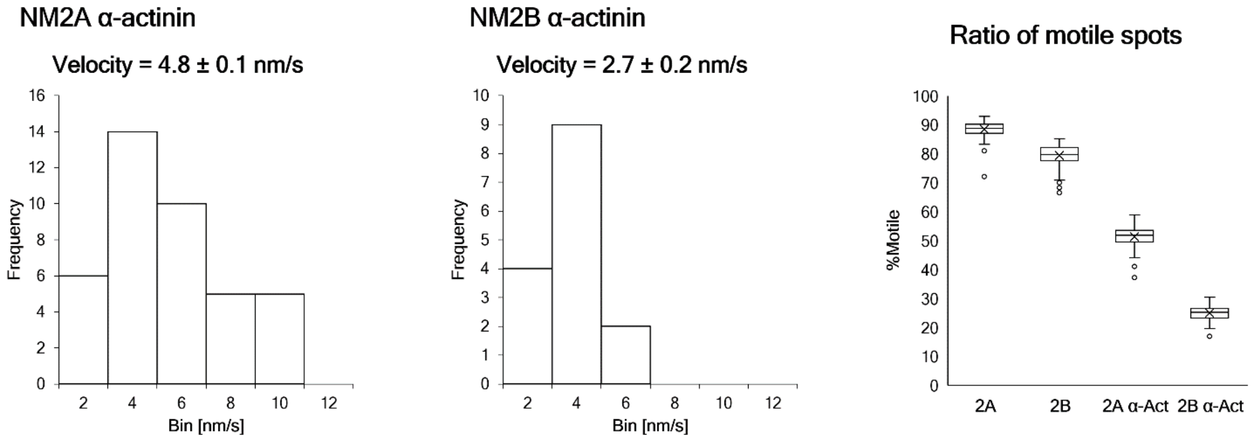

Supplementary Fig. 5: Motile properties of NM2 paralogs on sarcomeric actin patterns in the presence of α-actinin.

Histograms of NM2A (left) and NM2B (middle) representing the NM2 filament's velocity on sarcomeric actin patterns crosslinked by α-actinin. On the right the percentage of motile spots in each condition (NM2A, NM2B, NM2A with α-actinin and NM2B with α-actinin) is depicted, with each datapoint corresponding to 10 min segments of individual experiments. Together, the data shows that both the velocity and the overall motility of NM2 is impaired on antiparallel, crosslinked actin bundles.

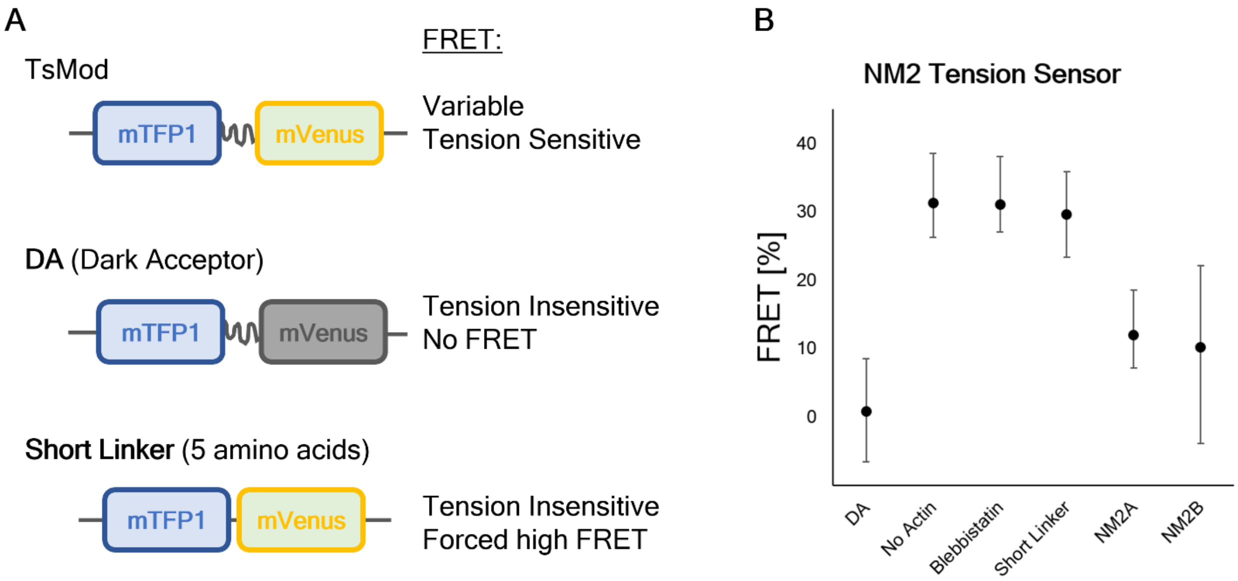

Supplementary Fig. 6: Tension sensor controls and corresponding FRET efficiencies determined via FLIM.

A The tension sensor module consists of a FRET donor (mTFP1) and acceptor (mVenus) connected by a flexible linker. When tension is applied, the linker gets stretched, reducing the FRET%, thus increasing the donor lifetime. The DA (dark acceptor) construct is tension insensitive due to an acceptor mutation, rendering it incapable of receiving energy via FRET. The short linker version is forced in a high FRET state, thus acting as a control for the sensor's relaxed state. **B** The NM2B DA construct reports a lifetime of 2.8 ns via FLIM (0% FRET), which is the longest possible lifetime measured. On the other hand, the NM2B short linker construct reports a lifetime near 2.0 ns corresponding to a corrected FRET efficiency of 30%, which fits other controls for the relaxed state, such as TsMod NM2B without actin and TsMod NM2B on patterned actin in the presence of para-Nitroblebbistatin. Two data points are added two show that TsMod NM2A and NM2B on patterned actin report lifetimes well between the two controls for the lower and upper limits.

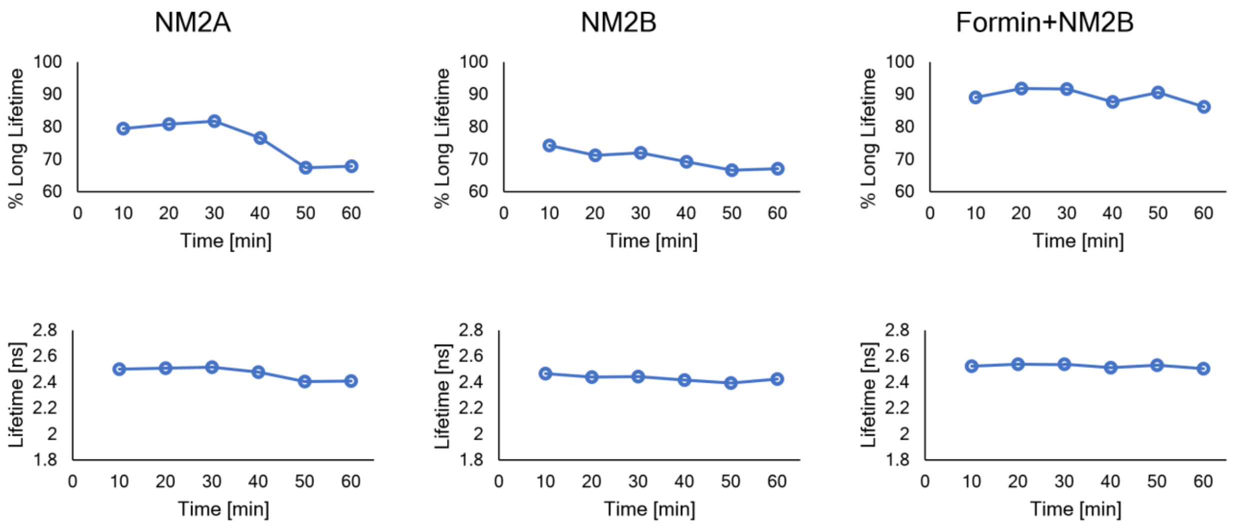

Supplementary Fig. 7: Quantification of tense vs relaxed motors within NM2 filaments using a global fitting algorithm.

As sarcomeric patterns mature, the tension sensor's lifetime determined via FLIM slowly drops for both NM2A, NM2B and the Formin tension sensor. As a global fitting algorithm is used to determine the long donor lifetime, it is possible to fit the ratio of the two main lifetimes within the FLIM signal. The lower lifetime hereby represents the relaxed fraction of molecules, while the long lifetime represents the tense state. While the tension produced by myosin heads is expected to lead to a variety of different tension levels during the crossbridge cycle, fitting for those two populations resulted in satisfying χ2 values, indicating that other states don't significantly contribute to sensor's signal. This analysis shows that around 70-80% of NM2s within a filament, and 90% of formins display the long lifetime, showing that this high fraction of molecules are on average in the tense state. During the course of the experiment, this fraction slightly drops, explaining a slight reduction of the reported lifetime.

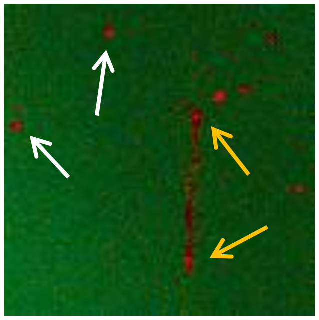

Supplementary Fig. 8: Representative trajectory of a TsMod NM2B filament sliding on a single actin filament via FLIM microscopy.

Single actin filaments functionalized with biotin-phalloidin are immobilized on a biotin-PEG surface using neutravidin. Filaments of TsMod-NM2B are then added to this surface to image their processive motility using FLIM microscopy. The image fit shows the background lifetime below 1.7 ns (green) and the relaxed lifetime belonging to TsMod NM2B near 2.0 ns (red). Both the filaments in the background that passively land on the surface (white arrows), as well as the filaments that land on a single actin filament (yellow arrows) and move processively (as shown by the trajectory) display the same lifetime that corresponds to 0 pN. This directly shows that NM2 filaments gliding on single actin filaments move virtually without load, unlike the tense filaments shown in a sarcomeric actin arrangement.

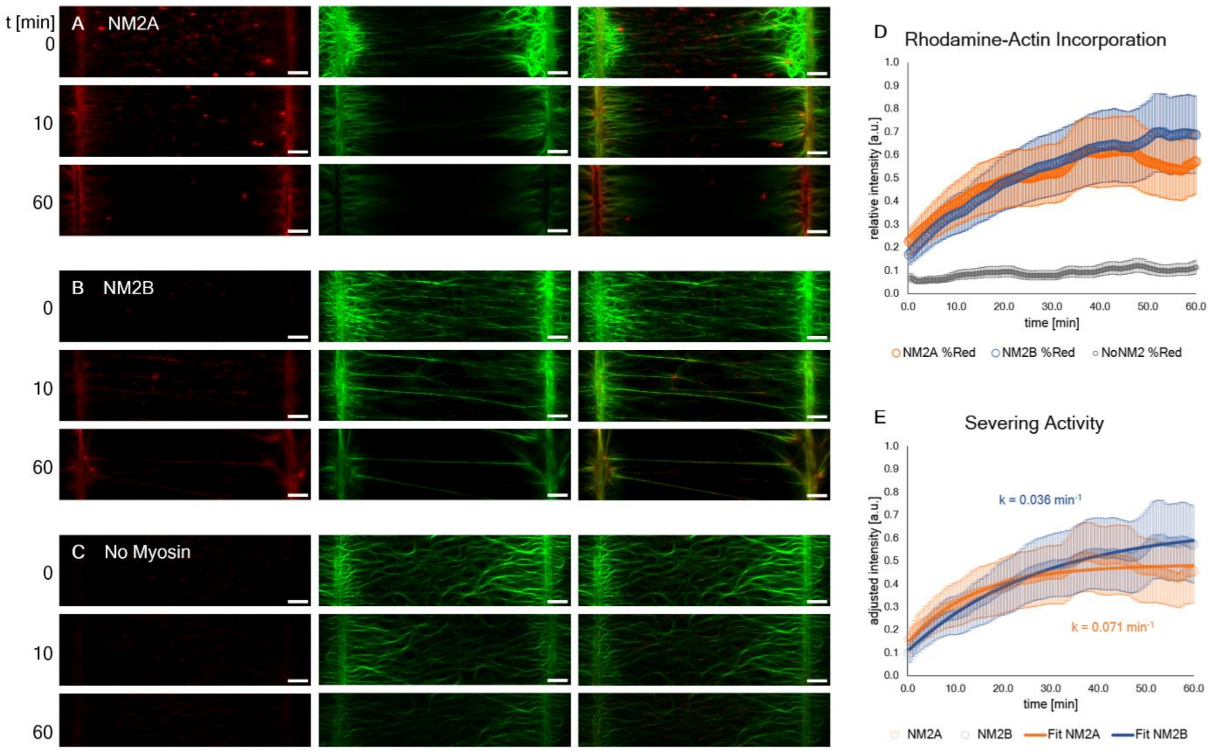

Supplementary Fig. 9: Quantification of the severing activity by barbed-end dependent actin incorporation.

To determine if either NM2A or NM2B are severing the actin filaments as they contract, sarcomeric patterns were polymerized in the presence of 488-phalloidin (green), after which the flow chambers were washed and covalently labeled rhodamine-actin monomers (red) were added in the presence of NM2A (**A**), NM2B (**B**) or no myosin (**C**). Addition of the red monomers is only expected at actin barbed ends, meaning it can be used to track the formation of barbed ends along actin bundles that form due to severing. The incorporation of the rhodamine actin was monitored over time via TIRF microscopy. The rhodamine label is shown in the left column, the phalloidin labeled pre-polymerized actin in the middle, and an overlay in the right column. The timestamps are in minutes, and 0 minutes represents the first measured frame, 2 minutes after adding the rhodamine-actin solution to the chamber. **D** The ratio of rhodamine actin to 488 actin at non-formin bound barbed ends over time. An increase can be observed for actin incorporation in the presence of both NM2A and NM2B, with a much slower incorporation without myosin. **E** To quantify the contribution of myosin activity to the generation of the barbed ends, both curves for NM2A and NM2B were normalized by the rate in the absence of myosin. Then, an exponential was fitted to calculate a severing rate of 0.071 min^{-1} for NM2A and 0.036 min^{-1} for NM2B. All scale bars are 10 µm.
